# Engineering Ultrasoft Interactions in Stiff All‐DNA Dendrimers by Site‐Specific Control of Scaffold Flexibility

**DOI:** 10.1002/smll.202308763

**Published:** 2024-01-06

**Authors:** Nataša Adžić, Clemens Jochum, Christos N. Likos, Emmanuel Stiakakis

**Affiliations:** ^1^ Faculty of Physics University of Vienna Boltzmanngasse 5 Vienna A‐1090 Austria; ^2^ Institute of Physics Belgrade University of Belgrade Pregrevica 118 Belgrade 11080 Serbia; ^3^ Institute for Theoretical Physics TU Wien Wiedner Hauptstraße 8–10 Vienna A‐1040 Austria; ^4^ Biomacromolecular Systems and Processes Institute of Biological Information Processing (IBI‐4), Forschungszentrum Jülich D‐52425 Jülich Germany

**Keywords:** dendrimers, DNA, DNA nanotechnology, flexible patches, self‐assembly, ultrasoft particles

## Abstract

A combined experimental and theoretical study of the structural correlations in moderately concentrated suspensions of all‐DNA dendrimers of the second generation (G2) with controlled scaffold rigidity is reported here. Small‐angle X‐ray scattering experiments in concentrated aqueous saline solutions of stiff all‐DNA G2 dendritic constructs reveal a novel anomalous liquid‐like phase behavior which is reflected in the calculated structure factors as a two‐step increase at low scattering wave vectors. By developing a new design strategy for adjusting the particle's internal flexibility based on site‐selective incorporation of single‐stranded DNA linkers into the dendritic scaffold, it is shown that this unconventional type of self‐organization is strongly contingent on the dendrimer's stiffness. A comprehensive computer simulation study employing dendritic models with different levels of coarse‐graining, and two theoretical approaches based on effective, pair‐potential interactions, remarkably confirmed the origin of this unusual liquid‐like behavior. The results demonstrate that the precise control of the internal structure of the dendritic scaffold conferred by the DNA can be potentially used to engineer a rich palette of novel ultrasoft interaction potentials that could offer a route for directed self‐assembly of intriguing soft matter phases and experimental realizations of a host of unusual phenomena theoretically predicted for ultrasoft interacting systems.

## Graphical abstract

A combined experimental and simulation work demonstrates that the intra‐structure patterning of a rigid dendritic scaffold with flexible patches at user‐prescribed positions can fine tune the bounded ultrasoft interactions among all‐DNA dendrimers of the second generation (G2).

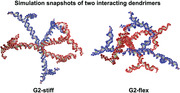

## 1 Introduction

Establishing a predictive relationship between the collective properties of large assemblies of building blocks and the effective interactions among them is an essential step for designing macroscopic functional soft materials. Different types of ultrasoft interaction potentials have received much attention in the last two decades due to their ability to stabilize novel bulk phase behavior of diverse states of matter,^[^ ^1^, ^2^, ^3^, ^4^, ^5^, ^6^, ^7^, ^8^, ^9^, ^10^, ^11^, ^12^, ^13^, ^14^, ^15^, ^16^, ^17^ ^]^ the most prominent of which is the recently experimentally discovered cluster crystal.^[^ ^18^ ^]^

To design interactions that promote self‐organization into specific equilibrium assemblies requires superior control of the building elements' chemistry and architecture. In this respect, DNA is a versatile nanoscale building block^[^ ^19^, ^20^ ^]^ owing to its programmable and fully addressable character, in combination with its unique physico‐chemical properties, which allow it to be easily modified and manipulated at the sub‐nanometre level (single base level).^[^ ^21^ ^]^ Over the last few decades, the remarkable growth of the research field of structural DNA nanotechnology has enabled the fabrication of static and dynamic DNA‐based nanoarchitectures of increasing complexity via the robust sequence‐complementarity of DNA strands,^[^ ^22^, ^23^, ^24^ ^]^ the shape‐complementarity based on the base‐stacking interactions between blunt‐ends of DNA helices^[^ ^25^ ^]^ and the site‐specific decoration of DNA structures with polymers and lipids.^[^ ^19^, ^26^ ^]^ This has allowed the emergence of novel applications^[^ ^19^, ^27^ ^]^ in diverse areas of research such as biophysics, biomedical engineering, plasmonics, drug delivery, and synthetic biology. Broadly speaking, the traditional engineering approaches focus on altering the building blocks' shape and surface chemistry.^[^ ^28^, ^29^, ^30^ ^]^ Recently, efforts have been shifted toward controlling a significantly less studied structural parameter, namely the internal flexibility of the building block. Control over this structural feature has already enabled the formation of novel crystalline phases, such as the folded smectic phase in all‐DNA chain‐sticks^[^ ^31^ ^]^ and cluster crystals in DNA‐based dendritic‐linear‐dendritic triblocks.^[^ ^18^ ^]^

In this vein, we exploit in this work the large difference in the persistence length *L*_p_ between double‐stranded DNA (dsDNA, $L_{p}^{\mathrm{dsDNA}}\approx \;50\;\mathrm{nm}$ in 0.15 m NaCl buffer)^[^ ^32^ ^]^ and single‐stranded DNA (ssDNA, $L_{p}^{\mathrm{ssDNA}}<2.0\;\mathrm{nm}$ in 0.15 m NaCl buffer)^[^ ^33^ ^]^ in order to engineer the scaffold flexibility of a stiff all‐DNA second‐generation (G2) dendrimer (G2‐stiff).^[^ ^34^, ^35^ ^]^ To achieve a flexible all‐DNA dendritic version (G2‐flex), we modify the internal structure of the G2‐stiff by incorporating ssDNA flexible spacers between adjacent generations (core and first layer). These DNA bridges consist of a poly‐T sequence, a choice motivated by the significantly reduced propensity of this sequence to form secondary structures.^[^ ^36^ ^]^ Key structural quantities of the solutions, namely the total scattering intensities and the structure factors obtained from a range of concentrations, demonstrate that the effective interactions between the dsDNA constructs are ultrasoft. At the same time, this ultrasoftness of the interaction can be further steered through the introduction of flexible bonds. We shall indeed demonstrate that such a structural modification generates a noticeable decrease in the internal rigidity of the dendrimer's scaffold without, however, altering notably its overall size. Through a combined theoretical and experimental study, we further show that the anomalous liquid‐like behavior as reflected in the calculated and measured structure factors emerging from moderately concentrated G2‐stiff dendrimers is governed by the scaffold's rigidity. Our results reveal that control over all‐DNA dendrimer's stiffness can lead to the realization of ultrasoft particles interacting via tunable repulsive, bounded pair potentials.

## 2 Results and Discussion

### 2.1 All‐DNA Dendritic Scaffold: Design and Modeling

**Figure 1:**
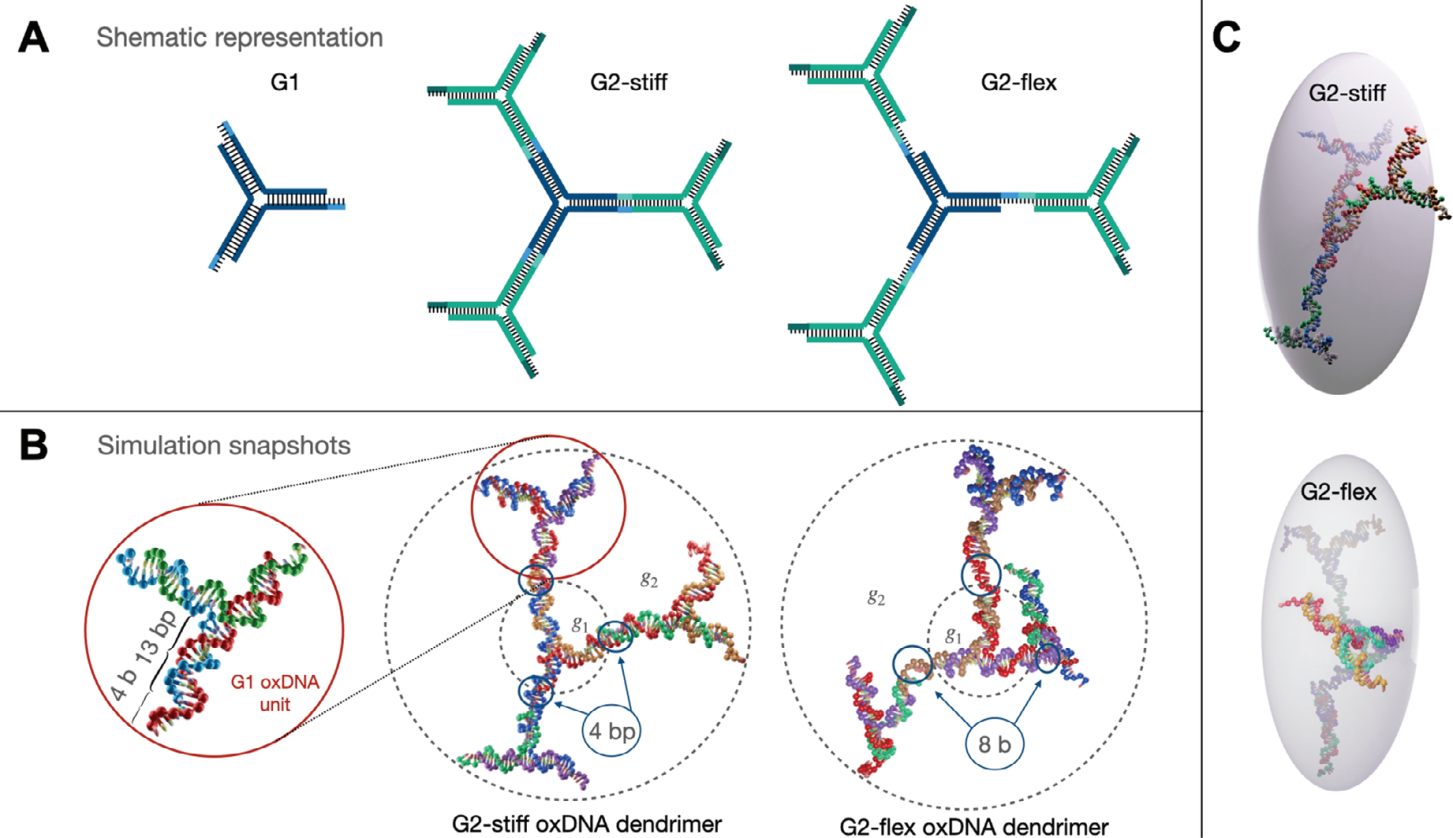
A) Schematic representations and B) simulation snapshots of all‐DNA Y‐shaped junction (G1) and G2 dendrimers. From left to right: Y‐shaped building block; G2‐stiff dendrimer with four base‐pair long rigid linkers between generations *g*_1_ and *g*_2_; and G2‐flex dendrimer with eight unpaired bases long flexible linkers between generations *g*_1_ and *g*_2_. C) Ellipsoid‐of‐gyration‐representation of G2‐stiff‐ and G2‐flex‐dendrimers. The oxView visualization tool^[^ ^38^, ^39^ ^]^ is employed for the demonstration of the all‐DNA structures.

A fully steerable self‐assembled all‐DNA dendritic nanostructure with high efficiency is fabricated solely from Y‐shaped all‐DNA (Y‐DNA) building blocks, employing a step‐by‐step assembly strategy. These three‐arm junctions are moderately flexible at the junction point^[^ ^37^ ^]^ and they consist of stiff, double‐stranded DNA (dsDNA) arms (thirteen (13) base‐pair (bp) long), which are terminated with two types of monofunctional single‐stranded DNA (ssDNA) dangling tails that are four bases long (4b), allowing other Y‐DNA to be covalently connected as each generation is added. A schematic representation of the fundamental Y‐DNA building blocks, and a simulation snapshot of the same are shown at the leftmost panels of **Figure** **1**A,B, respectively.

In this work, we restricted our studies to the second generation all‐DNA dendrimer (G2) with stiff and flexible connectors between the core (Y‐DNA, *g*_1_) and the first shell (*g*_2_). The G2 with stiff dendritic scaffold (G2‐stiff) was fabricated following synthetic protocols described in detail in ref. [35]. In short, a combination of a sticky ssDNA dangling tail and enzymatic ligation was used for the formation of 4‐bp‐long dsDNA stiff bridges between *g*_1_ and the *g*_2_ (Figure 1A,B). For the experimental realization of G2 with flexible dendritic scaffold (G2‐flex), we choose a non‐sticky ssDNA dangling tail terminated with azide or dibenzylcyclooctyne (dbco) moieties allowing a selective, fast and high‐yielding copper‐free click chemistry reaction^[^ ^40^ ^]^ between the *g*_1_'s Y‐DNA and the Y‐DNAs belonging to the *g*_2_ (more details in Experimental Section and Note S1, Supporting Information). This design leads to the creation of eight(8)‐bases‐long (8b) ssDNA bridges without essentially altering the dendrimer's total molecular weight (Figure 1A,B). This structural modification endows the G2 dendrimer with significant internal flexibility, as clearly demonstrated by the decreasing migration mobility of G2‐flex compared to the G2‐stiff in the polyacrylamide gel electrophoresis experiment, shown in Figure S1, Supporting Information. Strikingly similar flexibility‐dependent electrophoretic mobility is also observed in DNA with a linear topology.^[^ ^31^ ^]^ The dendrimers' free ends are passivated with poly‐T sequences (5T) to minimize aggregation due to base‐stacking interaction between blunt‐ended DNA helices.^[^ ^41^, ^42^ ^]^

The G2‐stiff and G2‐flexible all‐DNA dendrimers are modeled on the computer using oxDNA2^[^ ^43^ ^]^ (see Experimental Section for details). This is a coarse‐grained model that has been shown to capture the thermodynamic, structural, and mechanical properties of ssDNA and dsDNA. The basic building block of the model is a rigid nucleotide, and in this model, the DNA molecule is treated as a string of rigid nucleotides that interact through potentials depending on the positions and orientations of the nucleotides. In oxDNA2, the interaction between nucleotides takes into account sugar‐phosphate backbone connectivity, sequence‐dependent hydrogen‐bonding, excluded volume, and different types of stacking strengths (such as nearest neighbor stacking, cross‐stacking, and coaxial stacking between non‐nearest neighbors), including different interaction strengths for the AA and TT stacking. The latter is particularly useful for capturing the effects of stacking in ssDNA bridges in G2‐flex, since the poly‐T sequence is used as a flexible linker between *g*_1_ and *g*_2_. In addition, the oxDNA2 includes different widths for major and minor DNA grooves and a salt‐dependent interaction term, in the potential for modeling electrostatic interactions. The base sequences are chosen to match the ones used in experiments, and hydrogen bonding between complementary pairs of nucleotides on opposite strands can spontaneously occur, resulting in the formation of a B‐form double‐helix. Simulation snapshots of the above‐mentioned all‐DNA dendrimers using oxDNA2 are presented in Figure 1B; the blue circles in the snapshots indicate the location of the stiff and flexible bridges in the G2 scaffold, respectively.

In this work, we additionally assess the robustness of our simulation results regarding the conformation and unconventional structural correlations of our all‐DNA dendrimers by utilizing a bead‐spring (BS) model^[^ ^44^, ^45^ ^]^ (within the ESPResSo package),^[^ ^46^, ^47^ ^]^ which reduces significantly many of the degrees of freedom of the DNA nucleotide compared to the oxDNA2 model (more details about the BS model can be found in Note S2, Supporting Information). This type of coarse‐grained approach was successfully employed in previous work of ours in order to capture the conformational properties of isolated all‐DNA stiff dendrimers with generations ranging from G1 to G7.^[^ ^35^ ^]^ Most of the simulation work described here focuses on the oxDNA2 model simulation results, unless otherwise stated.

### 2.2 Conformational Behavior of Rigid All‐DNA Dendrimers with Controlled Internal Flexibility

**Figure 2:**
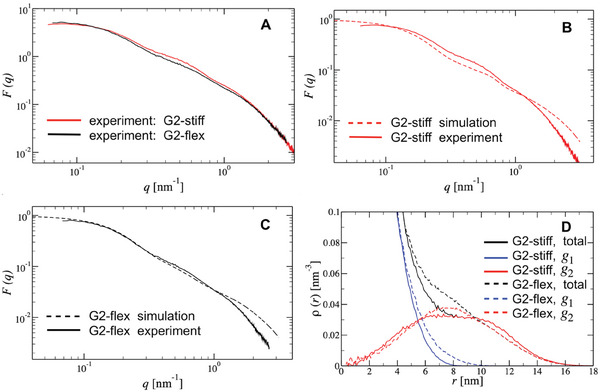
A) Form factor comparison between the G2‐stiff and G2‐flex measured in the experiment. B,C) Form factor comparison between oxDNA model and experiment for the G2‐stiff (B) and G2‐flex (C). D) Total density distribution function of nucleic acids, calculated within the oxDNA model for a single G2‐stiff (solid lines) and a single G2‐flex (dashed lines) dendrimer; together, the density distribution function of their corresponding subgenerations *g*_1_ and *g*_2_ is presented. The salt concentration is kept fixed at *c* = 0.15 m.

Aiming at obtaining a rigorous insight into the intramolecular structural signatures associated with the site‐specific positioning of flexible bridges within the stiff G2 scaffold, we perform a combination of small‐angle X‐ray scattering (SAXS) experiments in dilute aqueous solutions of G2‐stiff and G2‐flex molecules and molecular dynamics simulations of one isolated G2‐stiff and G2‐flex molecule based on the coarse‐grained oxDNA2 model at fixed salt (NaCl) concentration (c=0.15m). The measured and simulated G2‐stiff and G2‐flex form factors (FF) are shown in **Figure** **2**A–C.

The complete overlap of the experimental FF curves in Figure 2A, at the low scattering wavevector *q* region (*q* ⩽ 0.02 nm^−1^), demonstrates that the overall dimension of the G2 dendrimer is not essentially affected by the replacement of the stiff bridges between the *g*_1_ and *g*_2_ with flexible ones. However, the observed dissimilarities at the intermediate‐*q* region (0.02 nm^−1^ ⩽ *q* ⩽ 1.5 nm^−1^) highlight the effect of flexibility on the Y‐DNA's inter‐arm correlation within one single G2 dendrimer. While the FF of the G2‐stiff construct shows oscillations in this *q* region, in the G2‐flex case these are noticeable weaker, suggesting a more homogeneous monomer density at the *g*_1_ to *g*_2_ crossover. This effect can be ascribed to the back‐folding of the outermost Y‐DNA for the G2‐flex. The simulated FFs capture rather well the main features of the experimental ones (Figure 2B,C), with a systematic deviation at *q* > 1.0 nm^−1^. We attribute this discrepancy to the coarse‐grained level present in the oxDNA2 model, which deviates from the experimental system on such small length scales. The molecular properties related to the overall size and shape of the simulated G2‐stiff and G2‐flex at two different salt (NaCl) concentrations (0.15 and 0.5m) are summarized in the Experimental Section and in Table S1, Supporting Information. Both G2 molecules have similar sizes (*R*_g_≅9 nm) and a rather disk‐like shape, as can be seen from the corresponding ratios between gyration tensor eigenvalues and the relevant simulation snapshot in Figure 1C. In addition, G2‐stiff and G2‐flex demonstrate resilience in their shapes against increasing the salt concentration. Nevertheless, the data shown in Table S1, Supporting Information, suggests that the presence of the flexible bridges within the dendritic scaffold does result in a minor shrinkage and a tendency to deviate from the planar shape.

The quality of the comparison between simulations and experimental data offers us the possibility to exploit simulations to gain a deeper insight by looking at details of distributions and conformations within the dendritic scaffold. The internal structure of G2‐stiff and G2‐flex is quantified by obtaining the corresponding radial density distribution function ρ(*r*) of monomers as a function of the distance *r* from the center of the mass of the G2‐dendrimer. The total *g*_1_ and *g*_2_ monomer density profiles are depicted in Figure 2D. In both G2 molecules, the total monomer profile monotonically drops as one moves from the center of the molecule toward its periphery. However, in stark contrast to G2‐flex, the G2‐stiff exhibits a clear plateau at mid distances (6–10.5 nm), revealing a strong segregation between *g*_1_ and *g*_2_. This is also manifested in the density profile of monomers belonging to *g*_2_, which remains rather flat and constant at these inter‐particle distances. Accordingly, the G2‐flex features quite high local monomer packing in its interior compared to the G2‐stiff. In addition, the overlap region between the monomer density profiles of *g*_1_ and *g*_2_ is noticeably wider for the G2‐flex case, offering clear evidence that the flexible bridges in the G2 scaffold allow significant backfolding of the outer monomers toward the interior of the molecule.

Similar intramolecural correlations and overlap between subsequent subgenerations are also observed by employing the bead‐spring model, as evidenced by the computed total *g*_1_ and *g*_2_ monomer density profiles of G2‐stiff and G2‐flex systems presented in Figure S2, Supporting Information (see also relevant discussion in Supporting Information [Note S2, Supporting Information]). However, contrary to the oxDNA2 model, the corresponding *g*_2_ density profile shows a double‐peak feature, suggesting that the spatial distribution of *g*_1_ and *g*_2_ is more coherent. Most probably, the source of this discrepancy can be attributed to the overestimation of the Y‐DNA's arm stiffness and of the Y‐DNA's junction point flexibility (see the constants of bending energy in the BS model, Note S2, Supporting Information).

### 2.3 All‐DNA Dendrimers as Ultrasoft Colloids

Our previous work^[^ ^35^ ^]^ on the structural properties of all‐DNA stiff dendrimers at a single particle level with sizes ranging from G1 to G7 revealed that their well‐defined branched architecture and their inherent rigidity endow these nanostructures with low internal monomer concentration, regular voids in their interior, and, at the same time, a resilience against the addition of salt. These features, together with the DNA's highly charged character, allow us to view these complex macromolecular assemblies as penetrable ultrasoft colloids,^[^ ^48^ ^]^ and they are expected to have a significant impact on their collective behavior. In the following sections, we show results on unconventional structural correlations present in concentrated solutions of G2 molecules. Through a combined SAXS and computer simulation study based on effective potentials, the mechanism responsible for the observed anomalous structure factor behavior in the G2‐stiff system is identified.

#### 2.3.1 SAXS Apparent Structure Factors

**Figure 3:**
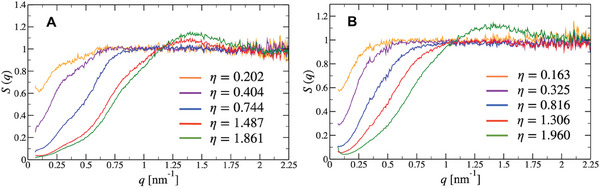
Experimental results for the structure factor *S*(*q*) of concentrated solutions of all‐DNA G2 dendrimers with A) rigid connections (G2‐stiff) and B) flexible connections (G2‐flex), at different packing fractions η with fixed salt concentration of *c* = 0.15 m.

Let us first define a dimensionless measure of dendrimer density by introducing the packing fraction η as

(1) $$\eta =\frac{4\pi N}{3V}R_{g}^{3}$$

where *N* is the number of dendrimers in the sample volume *V*, each molecule having a gyration radius *R*_g_. Measured static structure factors at room temperature for the G2‐stiff dendrimers are shown in **Figure** **3**A for concentrations below and above their overlap packing fraction. The apparent structure factor *S*(*q*), resulting from the interparticle interactions of the G2‐stiff molecules, is calculated assuming the validity of the relation *S*(*q*) = *I*(*q*)/*F*(*q*), where *F*(*q*) is the experimental form factor (Figure 2A) and *I*(*q*) is the measured SAXS intensity by the aqueous saline solutions (0.01m Tris, pH 8.0, 0.15m NaCl) of moderately concentrated all‐DNA dendrimers normalized to the volume fraction. It should be stressed that the factorization of structure‐ and form‐factors above is valid strictly only for systems whose constituent particles are rigid, that is, deprived of any internal fluctuations.

The structure factors of the stiff ds‐DNA dendrimer soltuions displayed in Figure 3A show the signature of ultrasoft effective interactions, which is the simultaneous suppression of the low‐*q* value of *S*(*q*) and the absence of a pronounced peak at finite *q*‐values. Indeed, for the usual, strongly repulsive effective interactions, the two effects appear together since low compressibility (a long‐wavelength property) is accompanied by the emergence of strong spatial correlations at finite length scales, whereas ultrasoft repulsive interactions result into incompressible systems lacking a pronounced spatial short‐range order.^[^ ^1^, ^49^, ^50^, ^51^ ^]^ For the case at hand, a peak indeed emerges, but the broad character of this peak and the corresponding spatial correlation value ($2\pi /q_{\mathrm{peak}}≃4.83\;\mathrm{nm}$) indicate the presence of just a weak liquid‐like positional order between overlapping G2‐stiff molecules. Furthermore, the presence of this noticeable correlation peak at a finite wavevector is accompanied by a decrease in osmotic compressibility, given by the *S*(*q*) → 0) limit, of the solution upon increasing the concentration. These characteristics are also present for the structure factors from concentrated flexible ds‐DNA solutions shown in Figure 3B; accordingly, both systems are governed by ultrasoft interactions. However, the introduction of flexible junctions allows for further, detailed manipulation of these interactions with visible effects in the form of structural correlations, as we will show below.

The most interesting part of the *S*(*q*) is the region of *q* ⩽ 1.2 nm^−1^, where for the G2‐stiff we observe a strikingly unexpected two‐step increase, see Figure 3A. This anomalous structure factor behavior seems to be present already at low packing fractions η of G2‐stiff, with the *S*(*q*) being more structured for η > 1.0 as evidenced by the appearance of a broad peak at *q*_peak_ ≃ 1.3 nm^−1^. To the best of our knowledge, the above‐mentioned peculiarity of *S*(*q*) at low scattering wavevectors *q* is absent in traditional neutral or charged dendrimers.^[^ ^52^, ^53^, ^54^, ^55^, ^56^, ^57^ ^]^ The distinctive difference between the low generation of these dendritic structures and our G2‐stiff dendrimers is that the former are flexible and the latter are rigid. Therefore, to shed light on the mechanism behind the non‐conventional behavior of the resulting *S*(*q*) in G2‐stiff molecules, their scaffold rigidity is reduced by altering the DNA structure at the interface between subsequent subgenerations. In the G2‐stiff case (mid‐panels in Figure 1A,B), the dsDNA stiff bridges between *g*_1_ and *g*_2_ are replaced by ssDNA flexible ones, leading to the formation of its flexible counterpart, which we will refer to as G2‐flex (the rightmost panels in Figure 1A,B). The corresponding concentration‐dependent static structure factors are shown in Figure 3B. They are calculated following a procedure similar to the G2‐stiff case, keeping the same ionic conditions. The results indeed reveal that, for sufficiently flexible bridges between subgenerations, the two‐step increase of *S*(*q*) disappears without affecting the liquid‐like broad peak at high packing fractions (η = 1.96). To gain a deeper insight to the basic mechanism responsible for the effect of dendritic scaffold rigidity on the stabilization of the aforementioned anomaly in the development of the structure factor, we derive and analyze an effective dendrimer interaction approach to predict the structural correlations of concentrated all‐DNA G2 molecules with controlled internal flexibility. Afterward, a direct comparison with experiments is feasible.

#### 2.3.2 Effective All‐DNA G2 Dendrimer Interactions

**Figure 4:**
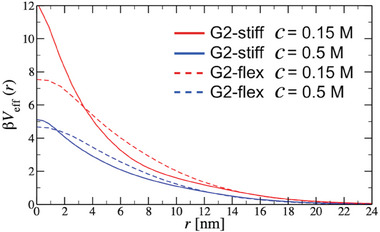
Effective pair interactions between two G2‐stiff (solid lines) and between two G2‐flex (dashed lines), calculated using Widom insertion method within oxDNA model and shown as a function of center‐of‐mass‐to‐center‐of‐mass separation *r*, at two different salt concentrations *c*.

An efficient way to study the collective organization of G2 molecules in concentrated solutions is to render them as effective particles, interacting with a coarse‐grained, effective potential that is calculated from the detailed interaction of two molecules under study. In order to calculate this effective interaction, the Widom insertion method is applied to the models of the G2‐stiff and G2‐flex system (see Experimental Section). In **Figure** **4**, the calculated effective interaction between two G2‐stiff dendrimers and as well as between two G2‐flex dendrimers at two different salt concentrations (c=0.15m and c=0.5m) is presented. All effective potentials are shown as a function of the distance between molecules' centers‐of‐masses. In marked contrast to the minor overall size difference between the G2‐stiff and G2‐flex molecules, these two systems have strikingly different behavior in terms of effective interactions. Similar behavior is also observed by employing the significantly less refined coarse‐grained BS model (see Figure S3, Supporting Information).

In Figure 4, it can be seen that all calculated potentials, besides being ultrasoft and short‐ranged, are also salt‐ and dendrimer‐type‐sensitive. The softness of potentials is a direct consequence of the all‐DNA‐dendrimer's fractal nature, which allows a non‐diverging interaction at overlapping separations. The short‐range character of the effective interactions is a result of the screened electrostatic interactions between charged DNA segments. In both models studied, these electrostatic interactions are incorporated through Yukawa screening potentials. This simplification is justified since both salt concentrations used in this work are high enough to allow the Debye–Hückel approximation.^[^ ^58^ ^]^ Therefore, the resulting effective interaction of G2‐stiff and G2‐flex dendrimers vanishes at interparticle distances slightly larger than 2*R*_g_≅18 nm and approaches zero with a Yukawa tail independent of the dendrimer type. Identical behavior is also observed in the bead‐spring model, as shown in Figure S3, Supporting Information.

These bounded, ultrasoft interactions can be additionally tuned by solution salinity. The effective potentials for both types of dendrimers at a salt concentration of c=0.5m (blue curves, Figure 4), are smaller in all inter‐dendrimer separations than the corresponding ones calculated at salt concentration of c=0.15m (red curves, Figure 4). This is rather expected since an increase in the salt concentration results in the screening of the Coulomb interactions between two like‐charged objects, yielding to a reduced repulsion. Finally, the observed decrease in effective interactions upon addition of salt becomes more enhanced as the inter‐dendrimer separation is decreased, indicating that electrostatics dominate over entropy.

The impact of the internal scaffold flexibility on the effective pair interactions of G2 dendrimers for a fixed salt concentration can be clearly seen in Figure 4. In the case of the dendrimers being in close proximity, the interpenetration of two G2‐flex molecules becomes more energetically costly than that of the G2‐stiff case. This is a direct consequence of the presence of significant fluctuations in the position and orientation of the outer arms of the G2‐flex system. In contrast to the G2‐stiff case, this internal flexibility permits the exploration of a wider range of available configurations when inter‐penetration begins. Further inter‐penetration of G2‐flex molecules is accompanied by a significant re‐orientation of their outer arms, adapting a more compact structure, which results in a higher energy penalty, whereas in G2‐stiff molecules, rigidity prevents significant changes in their conformation. Moreover, since the monomer density in *g*_2_ is low (Figure 2D), there is more available space for interpenetration without significant overlap of G2‐stiff's arms, resulting in a smaller energy penalty compared to the G2‐flex case. In the oxDNA2 model, the strength of the effective interaction at the complete overlap of molecules changes and becomes higher for the G2‐stiff (Figure 4), in contrast to the BS model case (Figure S3, Supporting Information). This energy difference between G2‐stiff and G2‐flex molecules becomes more pronounced at lower salt concentrations (*c* = 0.15~m). Apparently, at the complete dendrimer overlap state, the flexible bridges between the *g*_1_ and *g*_2_ in the charged G2‐flex molecule play a key role in minimizing the unfavorable monomer contacts at the inter‐dendrimer level and therefore the mutual electrostatic repulsion. This situation is clearly not captured by the BS model. However, despite this discrepancy, both the BS and the oxDNA2 model agree fairly well on the shapes and magnitudes of the potentials at both studied salt concentrations (*V*_eff_(0)≅5~*k*_B_ *T* at *c* = 0.5 m for G2‐stiff and G2‐flex and *V*_eff_(0)≅8~*k*_B_ *T* at *c* = 0.15 m for G2‐flex).

It is particularly important to emphasize that the presence of flexible bridges between *g*_1_ and *g*_2_ (G2‐flex system) has a marked effect on the resulting effective potential curves of all‐DNA G2 dendrimers (Figure 4). More specifically, the effective interactions of G2‐stiff molecules are initially changing slowly for large inter‐particle separations, and then as the separation becomes smaller than the dendrimers' radius of gyration, the curves become significantly steeper. This indicates the presence of two characteristic length scales in the effective interaction of G2‐stiff molecules, which is absent in the G2‐flex case. The microscopic origin of such a behavior can be traced back to the monomer density profiles of G2‐dendrimers, presented in Figure 2D. In contrast to the G2‐flex case, where the radial monomer density distribution function ρ(*r*) continuously increases as its center of mass is approached, the G2‐stiff case exhibits a stagnation at intra‐dendrimer distances between 6–11~nm. As a consequence, the interaction energy of G2‐flex molecules is larger than that corresponding to the G2‐stiff case in this range of interaction distances. Further approaching the center of the mass of the G2‐stiff leads to a faster growth of density distribution function (ρ(*r*) for *r* ⩽ 6nm in Figure 2D) which results in an abrupt increase of the interaction energy (*V*_eff_(*r*) for *r* ⩽ 6nm, see Figure 4). Our results, therefore, reveal that control over the internal flexibility of the dendritic scaffold can lead to the formation of dendrimers with tunable effective potentials, therefore controlling the shape of ultrasoft interactions via targeted flexibility of the intramolecular joints.

#### 2.3.3 Intermolecular Structural Correlations

The accuracy of the effective pair interaction model was tested in order to employ the calculated effective potentials to study the self‐organization of G2‐dendrimers in concentrated saline solutions. To achieve this, all‐molecules simulations of the dendrimer's solutions are required for calculating the radial distribution functions between the centers of mass, which in turn will be compared with the corresponding radial distribution functions extracted from the effective model. The effective model refers to a system of effective particles that interact through the corresponding effective potentials. This methodology is applied to the oxDNA2 and bead‐spring G2 dendrimer model, focusing only on the G2‐stiff effective interactions for different salt concentrations and packing fractions η, defined in Equation (1).

**Figure 5:**
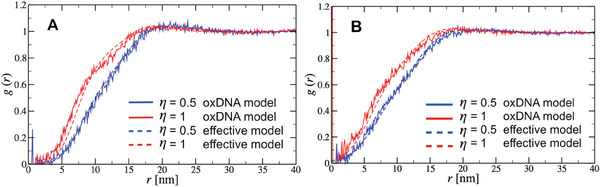
Radial distribution function comparison between explicit (oxDNA) and implicit (effective particles) MD simulations of solutions of G2‐stiff simulated for different values of packing fraction η and at two different salt concentrations: A) *c* = 0.15 m and B) *c* = 0.5 m.

The G2‐stiff radial distribution function *g*(*r*) of the explicit oxDNA2 model and the corresponding implicit effective model at two different salt concentrations is presented in **Figure** **5**. A similar comparison for the case of the bead‐spring model is shown in Figure S4, Supporting Information, where access to high dendrimer packing densities (η > 1) is attained. A remarkable quantitative agreement between the results of explicit and implicit simulations for η = 0.5 and η = 1.0 is found. At this point, it should be stressed that above the dendrimers' overlap packing fraction (η > 1.0) the many body effects raise serious concerns regarding the effective model validity for accurately predicting the system's collective organization. Previous studies in charged branched macromolecular assemblies with star‐like architecture^[^ ^59^ ^]^ suggested that three‐body forces become important when the star concentration exceeds at least 3–4 times the overlap density. The discrepancy between the radial distribution functions of explicit (BS model) and implicit simulations for η > 1.0 and salt concentration of *c* = 0.15 m in Figure S4, Supporting Information, can be likely ascribed to the above‐mentioned effect. In Figure 5, explicit *g*(*r*) demonstrates the emergence of a characteristic length, λ_*c*_≅8 nm, which corresponds to the position of a “shoulder” and gets more pronounced for higher packing fractions (Figure S4, Supporting Information). This feature is found in both salinities and matches nicely with the implicit simulation result for *c* = 0.5 m, however, it is not present in the implicit method for *c* = 0.15  m. Thus, precautions need to be taken when using the effective model to study highly concentrated all‐DNA dendrimers at low salinity conditions. In this particular case, the validity of the model will be tested by the comparison between simulation and experimental results; this is discussed in Section 2.3.5.

In Figure 5 and Figure S4, Supporting Information, the comparison of the simulation models (oxDNA2 and BS) and the calculated by the effective approach *g*(*r*) demonstrates that the latter methodology, which employs a simple Widom insertion approach, can provide reliable results for dendrimer concentrations below the overlap packing value (η = 1.0). However, the G2‐stiff radial distributions functions exhibit a behavior atypical for a normal liquid, in which the well‐defined peaks are absent and the shape of *g*(*r*) is reminiscent of an ultrasoft particles system. This weakly‐correlated‐liquid behavior is also encountered for η > 1.0. In addition, and independently from the coarse‐grained level of the employed explicit models, the *g*(*r*) shapes at packing fractions close to and above their overlap value reveal the presence of an unconventional structural organization that is more pronounced at the lower salt concentration (*c* = 0.15 m). This is evidenced by the development of a two‐step increase in *g*(*r*) for r<15.0nm, and indicates that G2‐stiff dendrimers exhibit an anomalous liquid‐like behavior.

Finally, it is imperative to note that for the effective interaction calculation, the isotropy of the potential was presumed when averaging the interaction energy over the solid angles. This is somewhat counter‐intuitive, since one would expect that this assumption is not valid for the anisotropic all‐DNA G2 dendrimers with a disk‐like shape (see Figure 1; Figure S5, Supporting Information, the simulation snapshots of the G2‐stiff BS model at two packing fractions η = 0.5 and η = 1.0). Nevertheless, the obtained consistency between the corresponding radial distribution functions in Figure 5 and Figure S4, Supporting Information, confirms the sufficiency of the used assumption. Moreover, when taking into account that those disks are hollow, formed by linear branches of DNA chains that change their orientation, the employment of a spherically symmetric pair potential between disk‐like objects is not a rough approximation but becomes rather more reasonable choice.

#### 2.3.4 Calculated Effective Structure Factors

**Figure 6:**
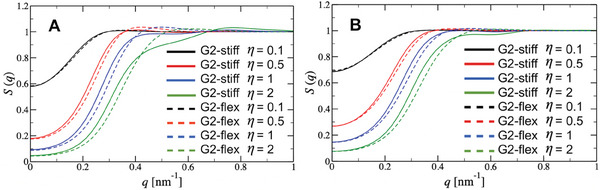
Structure factor of the solution of G2‐stiff (solid lines) and G2‐flex (dashed lines) calculated for different values of packing fraction η, at two different salt concentrations: A) *c* = 0.15 m and B) *c* = 0.5 m.

The spherically averaged static structure factor *S*(*q*) is calculated from the Fourier transform $\tilde{h}\left(q\right)$ of the pair correlation function *h*(*r*) = *g*(*r*) − 1 as $S\left(q\right)=1+\left(N/V\right)\tilde{h}\left(q\right)$. The results for increasing packing fractions of G2‐stiff and G2‐flex (0.1 ⩽ η ⩽ 2.0) at two different salt concentrations are presented in **Figure** **6** (*c* = 0.15 m in Figure 6A and *c* = 0.5 m in Figure 6B). All structure factors are calculated from the coarse‐grained model, employing the effective interaction calculated on the basis of the oxDNA2 model of G2 dendrimers. It is evident that for η ⩽ 1.0, the *S*(*q*) in both types of G2 dendrimers displays the usual liquid‐like behavior. The obtained structure factors at a salt concentration of *c* = 0.15 m exhibit a depression of the *S*(*q* → 0) value (Figure 6A), in agreement with a decrease in the osmotic compressibility of the solution upon increasing the concentration. As expected, an increase in salt concentration results in a more compressible system (Figure 6B, *c* = 0.5 m) due to the reduction of the inter‐dendrimers' Coulomb repulsion. Additionally, at low salt concentrations, a weak and broad but detectable correlation peak at a finite wavevector appears for dendrimer concentrations above their overall packing value (η > 1.0).

However, in contrast to the G2‐flex type of dendrimer, the G2‐stiff exhibits an anomaly in the development of the structure factor, namely a two‐step increase. Upon increasing the salt concentration, the scattering wavevector *q* at which this change in the increase of *S*(*q*) occurs is slightly shifted toward higher *q*‐ and *S*(*q*)‐values, as can be seen from the comparison between Figure 6A,B. This indicates that the screening of electrostatic interactions allows dendrimers to approach in closer proximity. At both salt concentrations and for packing fractions η > 1.0, these moderately concentrated G2‐stiff solutions are characterized by structure factors whose weak correlation peak corresponds to positional correlations in the range of 7–9 nm, while the *q*‐position of the reported anomaly indicates some level of self‐organization in the range of 13–15 nm.

The two well‐separated characteristic length scales mentioned above suggest the presence of two types of inter‐dendrimer configurations. The longer length scale corresponds to a weak liquid‐like positional order between neighboring G2‐stiff molecules; the shorter one, however, indicates dendrimer interpenetration at overlapping distances, which correspond to the plateau region in the total density profile of an isolated G2‐stiff dendrimer (Figure 2A), where a strong segregation between *g*_1_ and *g*_2_ takes place. This can be intuitively understood from the inherent rigidity of the dendritic arms and the low monomer density close to the dendrimer's center of mass. The energy penalty for the penetration of a G2‐stiff dendrimer with a neighboring one is not prohibitively large due to the dendrimer's open‐internal structure.

Once a small flexibility is introduced in the G2 system, the observed peculiar self‐organization disappears. Driven by the tendency to maximize entropy and avoid high levels of packing frustration, the molecules with flexible connections (G2‐flex) undergo shape and size fluctuations in the crowded environment, and therefore the configurations with such organized intertwined structures are prevented. Indeed, at high packing fractions, the G2‐flex system does not display such anomalous structure factor behavior but rather an infinite‐density ideal gas, where all correlations are smeared out due to the thermal fluctuations. Also, the maximum of the broad peaks in the corresponding *S*(*q*) (dashed lines in Figure 6) systematically move toward larger *q*‐values with increasing packing fraction, suggesting that the G2‐flex slightly shrinks. Finally, for the same packing fraction, the maximum of these peaks is located at larger *q*‐values compared to the G2‐stiff system, implying that upon introducing flexibility in the scaffold of the G2 dendrimer the average inter‐particle separation becomes smaller. The above mentioned impact of all‐DNA dendritic scaffold rigidity in the calculated effective structure factors is equally well captured with the significantly higher level of coarse‐gaining, BS model (see concentration‐ and salt‐dependence of *S*(*q*) in Figure S6, Supporting Information). Hence, this model independence in self‐organization prediction highlights the robustness of the calculated result regarding the anomaly in the structure factor of G2‐stiff and the physical origin of this unconventional behavior.

#### 2.3.5 Experiment Versus Simulation

The comparison between the concentration‐dependent structure factors *S*(*q*) from SAXS experiments (Figure 3) and simulations (Figure 6) at a salt concentration c=0.15m NaCl demonstrates that simulation results match fairly well the experimental trends on a qualitative basis. However, in quantitative terms, the anomaly in the development of the structure factor and the weak maxima in the experimental *S*(*q*) results occurred at noticeable higher values of the wavevector *q* compared to theoretical results. Furthermore, even though the correlation peak in *S*(*q*) appears for η > 1.0 in both experiments and simulations, its intensity is significantly larger in the measured case. Similar deviations are also observed between measured and BS‐based calculated *S*(*q*) (Figure 3A; Figure S6A, Supporting Information, respectively). It should be noted that not only the effective models but also the all‐particles‐resolved simulations (Figure 5) were unable to accurately predict the height and *q*‐position of the correlation peak in the measured *S*(*q*). Most of these discrepancies can be attributed to the factorization approximation of the scattering intensity, *I*(*q*) = *F*(*q*)*S*(*q*), the validity of which is subject to question^[^ ^60^ ^]^ once we approach the dendrimers' overlap concentration and diminish the rigidity of the dendritic scaffold (particle deformability). Therefore, in order to avoid a direct yet misleading comparison between these apparent and simulated structure factors from concentrated solutions of G2‐stiff and G2‐flex dendrimers, a comparison of scattering intensities has to be performed for densities below their overlap value.

**Figure 7:**
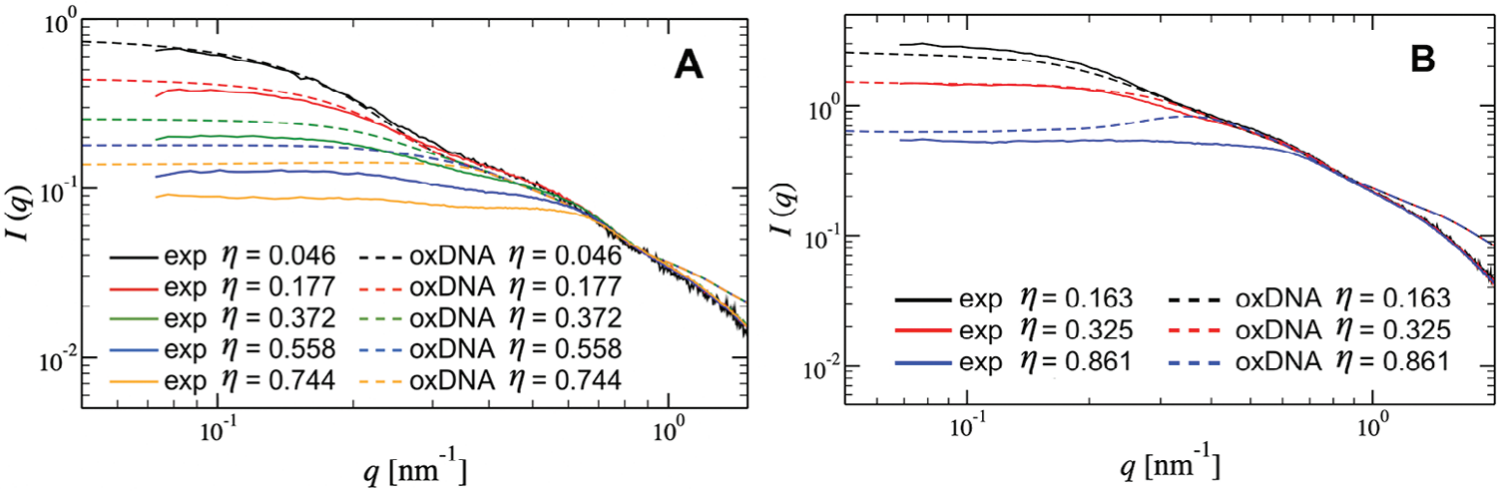
A comparison between total scattering intensities *I*(*q*) normalized to the volume fraction η as function of *q* from experimental (exp) and simulated (oxDNA) A) G2‐stiff and B) G2‐flex systems, at salt concentration *c* = 0.15 m.

In **Figure** **7**, the experimentally measured and theoretically calculated scattering intensities of the G2‐stiff and G2‐flex dendrimers normalized to volume fraction as a function of *q* are displayed. The latter are obtained by applying the integral equation theory using the effective potential approach. The structure factor of the G2‐stiff and G2‐flex systems is extracted using the oxDNA2 model and the corresponding effective potentials (Figure 4). Together with the calculated form‐factor from infinite dilution (Figure 2B,C), the theoretically scattering intensity is derived by the factorization formula. In the G2‐stiff case, the agreement between intensities measured in the experiment and calculated in the simulation is excellent for the two lower packing fractions (η < 0.2) (Figure 7A). However, the onset of the discrepancy in the *q*‐position of the peak in the scattering profiles starts to be detectable as the overlap density is approached. For packing fractions in excess of η = 0.5, an additional discrepancy regarding the osmotic compressibility is observed, indicating that the repulsion between G2‐stiff molecules is significantly underestimated by the simulations. This is an indication that at this packing fraction, when crowding starts, the entropic contribution prevails, short‐range, strongly repulsive steric interactions between the DNA strands are not taken into account properly by the oxDNA‐model. For *q* ⩾ 1.0nm^−1^, simulations fail to describe the measurements, most probably due to the coarse‐grained level of the ox‐DNA2 model, which affects the calculated form‐factor (see also Figure 2C,D for *q* ⩾ 1.0nm^−1^). Finally, it can be seen in Figure 7B, that a similar scattering intensity comparison between experiments and simulations is also present in the G2‐flex system upon increasing the dendrimer's density. However, in contrast to G2‐stiff, a very satisfactory agreement between measured and calculated compressibilities is observed, up to packing fractions of η = 0.816.

## 3 Conclusions

In this work, the impact of scaffold rigidity on ultrasoft interactions among all‐DNA dendrimers was specifically assessed. In contrast to traditional dendrimers, the all‐DNA dendritic construct is a unique, highly charged, void‐containing macromolecular assembly^[^ ^35^ ^]^ which is afforded by the stiff character of the short dsDNA fragments consisting of the Y‐DNA; the only building block from which these dendrimers are built. Through a combined experimental and simulation study, the structural organization of moderately concentrated solutions of all‐DNA G2 dendrimers with stiff (G2‐stiff) and with flexible patchy (G2‐flex) internal structure was contrasted. The key structural investigation here has entailed the precise control of dendritic scaffold rigidity via the insertion of flexible single‐stranded DNA domains between the core and the shell and then examining their role in the dendrimers' self‐organization behavior.

Unambiguous evidence for the emergence of a novel anomalous liquid‐like organization in concentrated aqueous solutions of G2‐stiff dendrimers is provided. This is experimentally demonstrated as a two‐step increase in the measured structure factors *S*(*q*) as extracted from SAXS measurements. Site‐specific weakening of dendrimer's scaffold rigidity, resulting in the fabrication of the G2‐flex dendrimer, is found to be the key parameter for the complete suppression of this type of structural correlation pinpointing under what conditions this anomaly in the development of the structure factor happens. The experimental findings are in good, semi‐quantitative agreement with simulations, despite the notable discrepancy regarding the *q*‐position of this structural anomaly in *S*(*q*). The latter seems to be associated with overestimation of the range of the electrostatic interactions in the simulated concentrated solutions of these charged all‐DNA dendrimers, resulting in an observed overestimation of the relevant length scale. It nonetheless transpires that the extensive computer simulations performed, employing two G2 dendritic models with different levels of coarse‐graining, demonstrate the validity of this unconventional type of self‐organization and enable us to identify the interaction mechanisms that are key to the formation of the reported anomaly in the structure factor of G2‐stiff dendrimers.

In particular, the structure factor behavior in the G2‐stiff and G2‐flex systems, which in turn encodes the effective interaction potential between the dendrimers, allows us to clearly distinguish these two types of ultrasoft particles despite the seemingly small internal structural difference. The G2‐flex interaction follows a Gaussian core model scenario,^[^ ^49^ ^]^ which is driven by the properties of the bounded effective interactions at overlapping distances and follows only one scaling law. However, the G2‐stiff interaction, besides being bounded, is also characterized by two length scales, which result in an anomalous structure factor above the overlap concentration. In the context of polymer stars, it has been proven that the existence of two length scales in the interaction energy is the cause of an anomalous structure factor in which the second peak is higher than the first one (see refs. [59, 61, 62]). However, in contrast to the polymer star case, the all‐DNA G2 dendrimers experience a bounded interaction, and therefore the system at hand is unique in terms of effective interactions and self‐organization behavior.

The intra‐structure patterning of a particle with flexible patches at user‐prescribed positions could substantially enrich the emerging bottom‐up programmable self‐assembly platform based on the control of directionality in the non‐covalent interactions between the constituent particles of a self‐assembled system. Here, the above self‐assembly concept is demonstrated for the all‐DNA dendrimer case. The reported synthetic scheme is quite robust and modular since DNA permits profound control over the position and distribution of the flexible single‐stranded domains within the dendritic scaffold with sub‐nanometer precision. The most prominent examples include the combination of two different Y‐DNA building blocks in tandem, one type terminated with sticky ends and the other with biorthogonal reaction pairs from the copper‐free click‐chemistry toolbox, to realize all‐DNA dendrimers with alternate flexible‐to‐rigid shells. In a similar fashion, combining these two different types of terminal reactivity in a single Y‐DNA building block could lead to the development of a new type of dendritic‐based Janus particles with distinct softness faces. Further work on exploring the utility of all‐DNA dendritic scaffolds toward these endeavors could pave the way to the engineering of novel ultrasoft interaction potentials that are absent in conventional soft‐matter particles with isotropic softness.

## 4 Experimental Section

### Synthesis and Characterization of All‐DNA G2‐Stiff and G2‐Flex Dendrimer

Custom and modified (5′‐phosphate, 5′‐azide‐PEG4, 5′‐dbco‐PEG4) oligonucleotides were purchased from Biomers (www.biomers.net) and purified by HPLC. The DNA concentration was determined by measuring the absorbance at 260 nm with a micro‐volume spectrometer (NanoDrop 2000). Following the synthetic protocol described in ref. [35] all‐DNA G2‐stiff dendrimers were fabricated from double‐stranded, three‐armed Y‐shaped DNA (Y‐DNA) with sticky ends by combining a step‐by‐step base‐pairing and enzyme‐assisted assembly strategy. A strain‐promoted alkyne‐azide cycloaddition (SPAAC, copper‐free click chemistry) reaction^[^ ^40^ ^]^ was employed for the fabrication of the G2‐flex construct, using adide‐ and dbco‐functionalized Y‐DNA building blocks. The optimal ratio of reacting compounds was determined based on the protocols that were developed in ref. [26]. In the G2‐flex case, the optimal ratio at which the electrophoresis experiment did not show any evidence of unreacted azide‐Y‐DNA was the mixing of dbco‐Y‐DNA and azide‐Y‐DNA in ratios of 7:1. The excess dbco‐Y‐DNA was removed using dialysis (Spectra/Pro, Float‐A‐Lyzer G2 dialysis device with molecular weight cutof off 100 kDa). DNA sequences and construction schemes for synthesizing the all‐DNA G2‐stiff and G2‐flex dendrimers are presented in Note S1, Supporting Information. Native polyacrylamide gel electrophoresis (PAGE) experiments were used to characterize the self‐assembled G2‐stiff and G2‐flex dendrimers, an example of which is shown in Figure S1, Supporting Information. Only one band for each Y‐DNAs building block involved in the dendrimer construction was detected, suggesting that they were properly formed. The desired all‐DNA dendritic constructs migrated as single, sharp bands, with the G2‐flex demonstrating undoubtedly slower mobility compared to the G2‐stiff.

### Experimental Systems Parameters

The all‐DNA G2‐stiff and G2‐flex packing fraction η was estimated, in full agreement with its definition in Equation (1) for the theoretical approach, using the following expression

(2) $$\eta =c_{\mathrm{DNA}}\frac{4\pi N_{A}}{3M_{w}}R_{g}^{3}$$

where *c*_DNA_ is the mass density of dendrimers, *M*_w_ represents the molecular weight of the dendrimer, in particular $M_{w}^{G2-\mathrm{stiff}}=112.67\;\mathrm{KDa}$, $M_{w}^{G2-\mathrm{flex}}=116.44\;\mathrm{KDa}$, *R*_g_ the radius of gyration from oxDNA‐simulations ($R_{g}^{G2-\mathrm{stiff}}=9.4\;\mathrm{nm}$, $R_{g}^{G2-\mathrm{flex}}=9.1\;\mathrm{nm}$), and *N*_A_ is Avogadro's number. The concentrated dendrimer solutions were prepared using Tris‐HCl buffer (10mm), pH 8.0 with 150mm NaCl.

### Small‐Angle X‐Ray Scattering (SAXS)

SAXS experiments were carried out at the high brilliance galium anode low angle X‐ray instrument (GALAXI) of the Jülich Center for Neutron Science (JCNS, Germany).^[^ ^63^ ^]^ A Dectris–Pilatus 1M detector with a resolution of 981 × 1043 pixels and a pixel size of $172\times 172\;\mu m^{2}$ was employed to record the 2D SAXS scattering patterns. The samples were measured at a sample‐to‐detector distance of 1.706 m with a flux of 10^9^ photons/s/mm^2^ at 0.3 mrad divergence of the beam at the sample position. The 1D‐SAXS profiles were obtained by azimuthally‐averaged radial scattering intensity of the 2D‐SAXS images using the FIT2D software. The solutions were loaded into borosilicate glass capillaries (Hilgenberg) with a thickness of 2.0 mm.

### oxDNA Simulations

Coarse‐grained molecular dynamics simulations (MD) were carried out with the oxDNA2 simulation platform.^[^ ^43^, ^64^ ^]^ To generate the G2‐stiff and G2‐flex dendrimer configurations, the experimental oligos sequences (Note S1, Supporting Information) were used as input. The single‐stranded oligos were hybridized by applying a harmonic potential between complementary bases. A canonical ensemble simulation setup was employed at room temperature 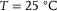 with periodic boundary conditions and a fixed Brownian thermostat. In the MD simulations, the timestep was set to Δ*t* = 0.001 with a total of 10^9^ steps, corresponding to a real time of 3.03 × 10^−6^ s.

### Effective Interaction Simulations

To accelerate and save computational time when studying the all‐DNA‐dendrimer solutions, the Widom insertion method^[^ ^65^ ^]^ is employed to calculate effective interactions between two G2‐stiff allDNA dendrimers as well as between G2‐flex allDNA dendrimers. For that purpose, a single dendrimer was equillibrated and inserted into a large box in a fixed position. Another dendrimer of the same kind was added, having an uncorrelated configuration with the first one. The MD simulations were then performed keeping one dendrimer fixed while inserting the other one a large number of times, in particular for a total of 5 × 10^6^ Widom insertions, to sample all possible random configurations and orientations for the wide range of center‐of mass to center‐of‐mass separations. Let now $s_{i}^{M}$, *i* = 1, 2 denote the collective coordinates of the *M* monomers of dendrimers 1 and 2, and $V_{12}\left(s_{1}^{M},s_{2}^{M};r\right)$ denote the microscopic interaction potential between the monomers of the two different dendrimers at the instantaneous conformations $s_{1}^{M}$ and $s_{2}^{M}$, under the constraint that their centers of mass have a separation *r*. The effective potential *V*_eff_(*r*) is obtained as^[^ ^65^ ^]^

(3) $$V_{\mathrm{eff}}\left(r\right)=-k_{B}T\ln{\langle e^{-\beta V_{12}\left(s_{1}^{M},s_{2}^{M};r\right)}\rangle }_{0}$$

where the averaging is performed over a non‐interacting Hamiltonian between the two dendrimers. The obtained potential was then used as an effective interaction energy between point particles. The system of point particles interacting with the given potential was employed to mimic the solution of G2‐stiff and G2‐flex all‐DNA‐dendrimers.

## Author Contributions

N.A. and C.J. performed simulations. C.N.L. co‐designed research and supervised the work. E.S. co‐designed the research and performed synthesis and experiments. All authors wrote the paper and commented on the manuscript.

## Conflict of Interest

The authors declare no conflict of interest.

## Supporting information

Supporting Information

## Data Availability

The data that support the findings of this study are available from the corresponding author upon reasonable request.
