# Supplementary material for: Engineering Ultrasoft Interactions in Stiff All‐DNA Dendrimers by Site‐Specific Control of Scaffold Flexibility
Source: Small. 2024 Jan 6;20(21):2308763. doi: 10.1002/smll.202308763 (PMC11475228; doi:10.1002/smll.202308763)
Supplement: Supplementary file 1 — Supporting Information [file SMLL-20-2308763-s001.pdf]

NANO · MICRO
small

Supporting Information

for *Small*, DOI 10.1002/smll.202308763

Engineering Ultrasoft Interactions in Stiff All-DNA Dendrimers by Site-Specific Control of Scaffold Flexibility

Nataša Adžić, Clemens Jochum*, Christos N. Likos* and Emmanuel Stiakakis**

Supporting Information

Engineering ultrasoft interactions in all-DNA dendrimers by site-specific controlling their scaffold flexibility

Nataša Adžić, Clemens Jochum, Christos N. Likos and Emmanuel Stiakakis

Contents

Supporting Figures S1-S6

Supporting Table S1

Supporting Note 1: All-DNA G2 construction schemes

Supporting Note 2: Bead-spring (BS) model

References

Supporting Figures

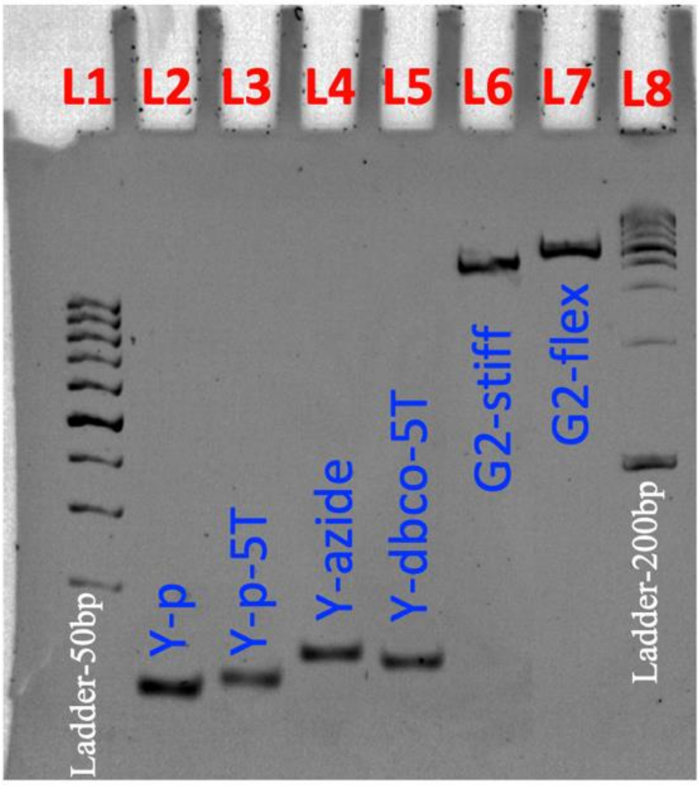

Figure S1. Native polyacrylamide gel electrophoresis (PAGE-5%) analysis of the construction of G2 all-DNA dendrimers with controlled scaffold flexibility. Lane 1: 50 base-pair (bp) DNA ladder (Jena Bioscience), from bottom: 100bp-to-500bp with 50bp step; Lane 2: Y-DNA belonging to g_1 sub-generation with the 5'-end of each DNA arm phosphorylated (Y-g_1-stiff); Lane 3: Y-DNA belonging to g_2 sub-generation with the 5'-end of only one DNA arm phosphorylated (Y-g_2-stiff); Lane 4: Y-DNA belonging to g_1 sub-generation with the 5'-end of each DNA arm functionalized with an azide moiety (Y-g_1-flex); Lane 5: Y-DNA belonging to g_2 sub-generation with the 5'-end of only one DNA arm functionalized with a dibenzylcyclooctyne (dbco) moiety (Y-g_2-flex); Lane 6: Purified G2-stiff consisting of one Y-g_1-stiff and three Y-g_2-stiff; Lane 7: Purified G2-flex consisting of one Y-g_1-flex and three Y-Y-g_2-flex; Lane 8: 200 base-pair (bp) DNA ladder (Jena Bioscience), from bottom: 200bp-to-2000bp with 200bp step.

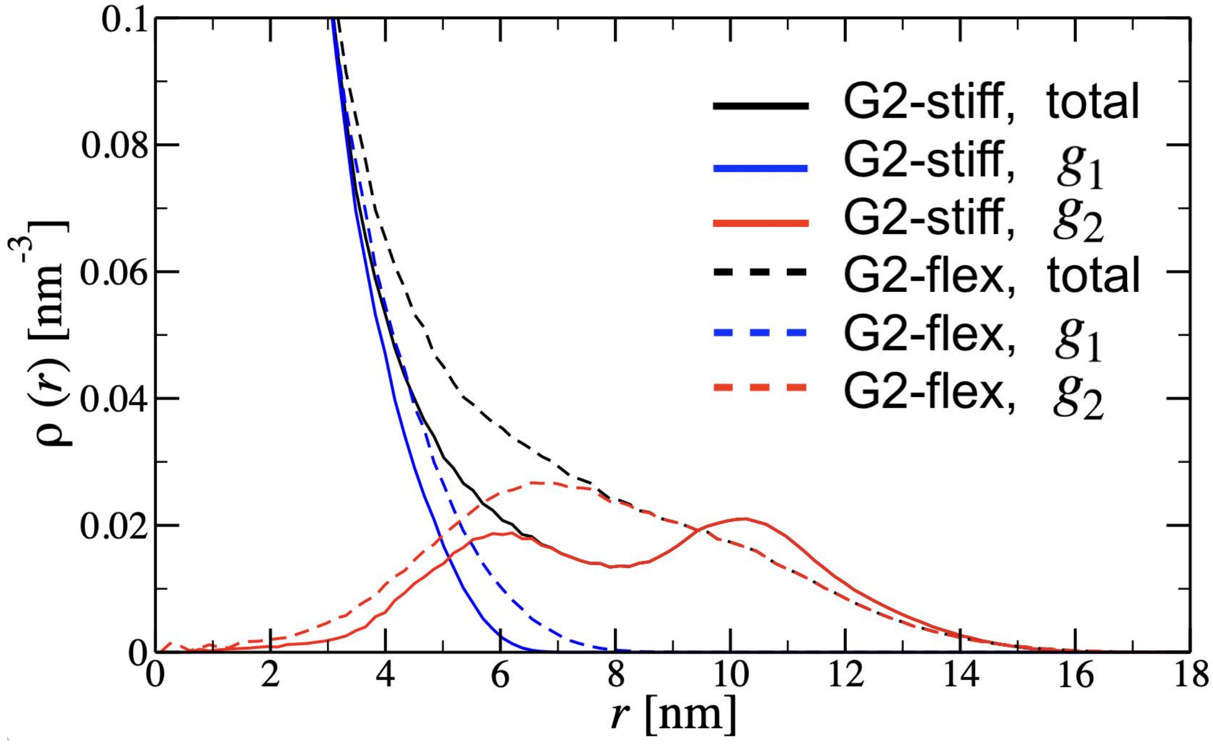

Figure S2. Density distribution function of monomers, calculated within bead-spring model, for a single G2-stiff (solid lines) and a single G2-flex (dashed lines). Together their corresponding sub-generations g_1 and g_2 are also presented. The salt (NaCl) concentration is kept fixed at c = 0.15 M.

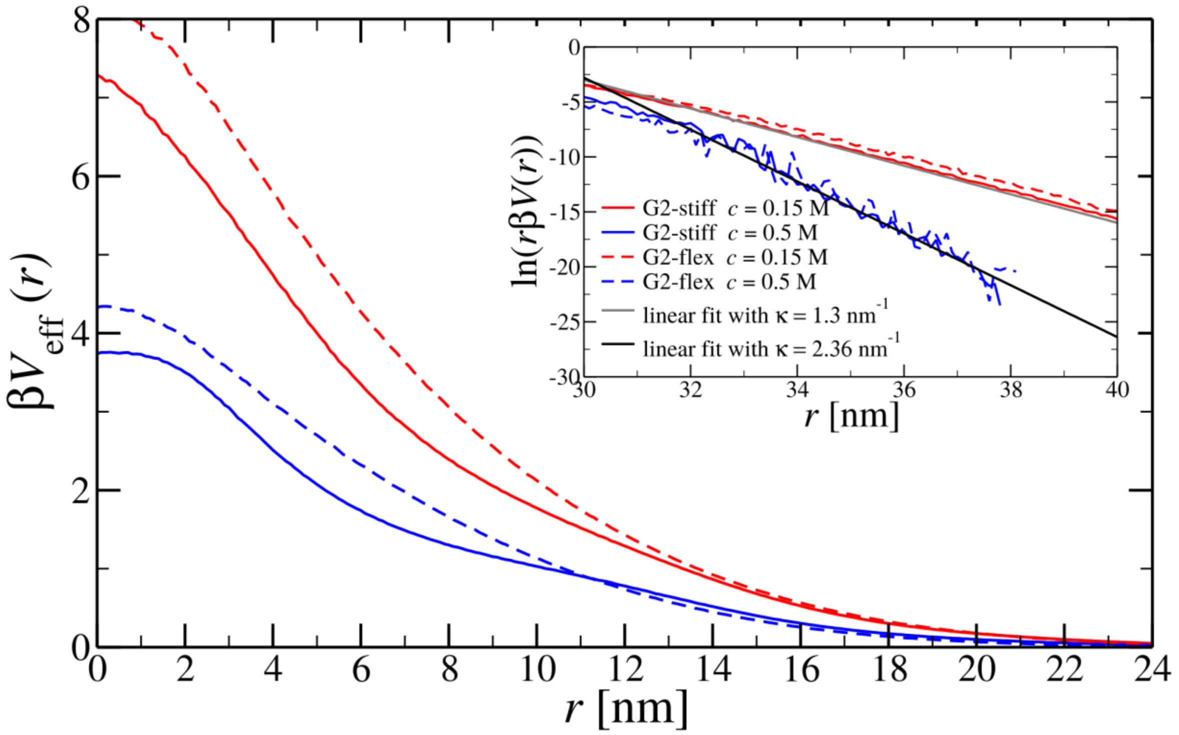

Figure S3. Effective pair interactions between two G2-stiff (solid lines) and between two G2-flex (dashed lines), calculated using Widom insertion method within the bead-spring model and shown as a function of center-of-mass-to-center-of-mass separation r, at two different values of salt (NaCl) concentrations c. The inset shows that at larger separations the effective potentials turn into the pure Yukawa interaction with Debye-screening lengths corresponding to the given salt concentrations.

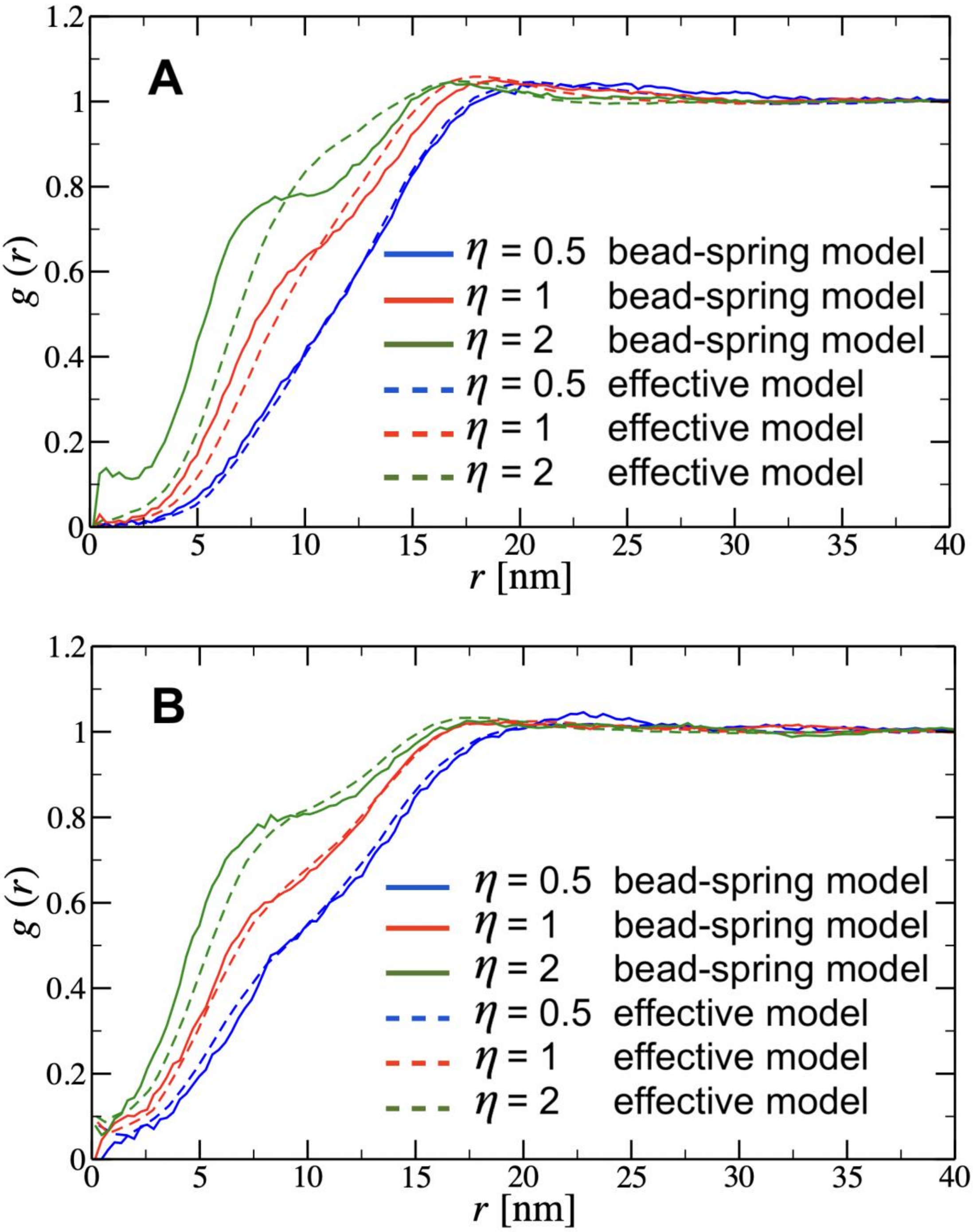

Figure S4. Radial distribution function comparison between explicit (bead-spring) and implicit (effective particles) MD simulations of solutions of G2-stiff simulated for different values packing fraction η and at two different values of salt concentration: A) $c = 0.15$ M and B) $c = 0.5$ M.

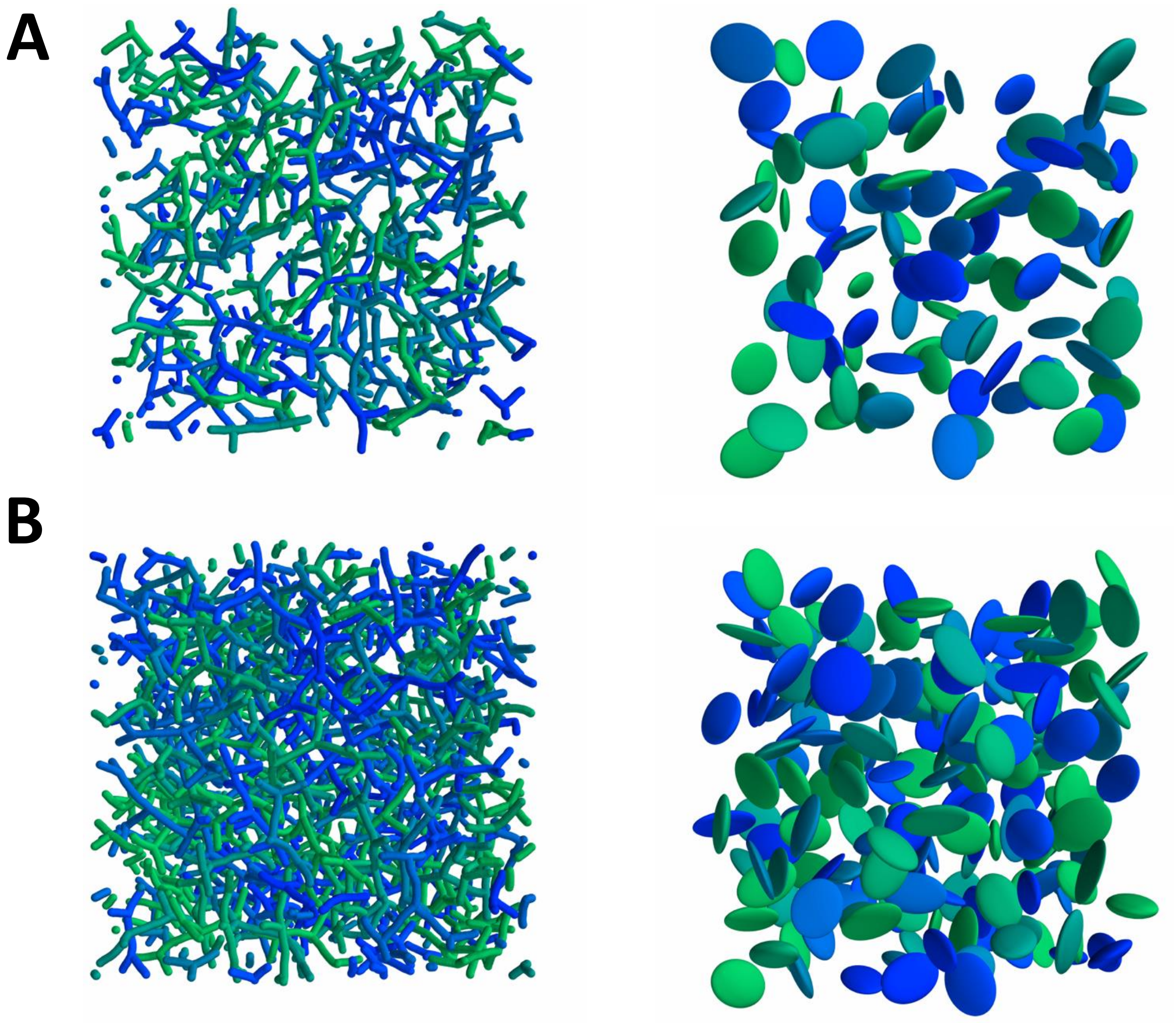

Figure S5. Simulation snapshots of bead-spring G2-stiff dendrimers at two different packing fractions: A) $\boldsymbol{\eta = 0.5}$ B) $\boldsymbol{\eta = 1.0}$ The corresponding visual representations of radius gyration tensor of the molecules in the solutions are also presented (left snapshots). The salt (NaCl) concentration is kept fixed at c = 0.15 M.

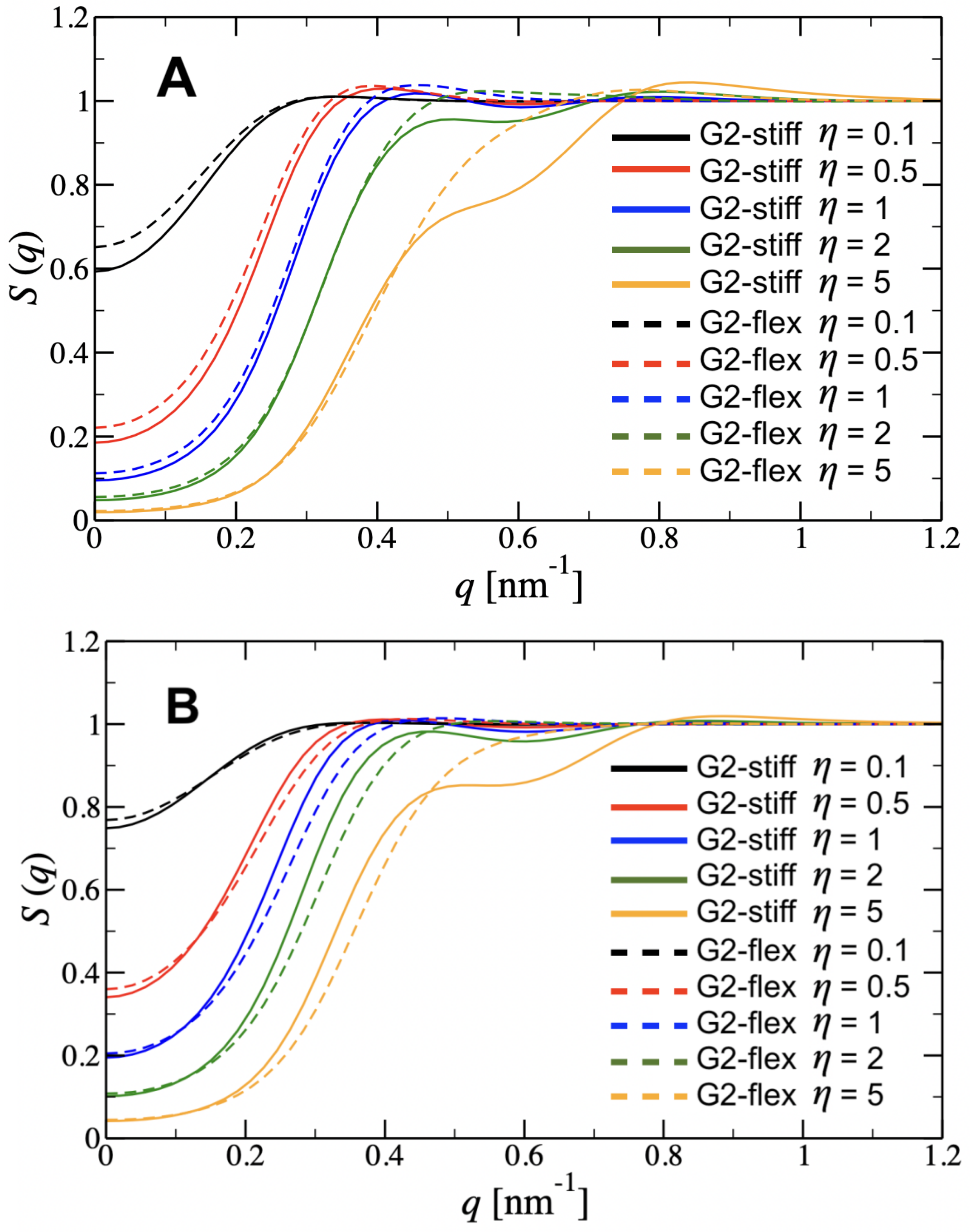

Figure S6. Structure factor *S(q)* of the solution of bead-spring model G2-stiff (solid lines) and G2-flex (dashed lines) calculated for different values of packing fraction η, at two different salt concentrations: A) $c = 0.15$ M and B) $c = 0.5$ M.

Tables

Table S1: The properties of a single all-DNA G2-stiff and G2-flex dendrimer molecule are presented; N stands for the number of monomers (bead-spring model) or amino acids (oxDNA model) and $R_g(c = 0.15M)$, $R_g(c = 0.5M)$ refer to the radius of gyration at the given value of salt (NaCl) concentration c. Together, the corresponding ratios between gyration tensor eigenvalues $\lambda_1^2 > \lambda_2^2 > \lambda_3^3$ are given for both salt concentration considered. The **Figure S5** is produced using the corresponding values of gyration tensor.

All-DNA G2 model	Type	N	$R_g(c = 0.15M)[nm]$	$R_g(c = 0.5M)[nm]$	$\frac{\lambda_2^2}{\lambda_1^2}(c = 0.15M)$	$\frac{\lambda_3^2}{\lambda_1^2}(c = 0.15M)$	$\frac{\lambda_2^2}{\lambda_1^2}(c = 0.5M)$	$\frac{\lambda_3^2}{\lambda_1^2}(c = 0.5M)$
BS	G2-stiff	192	9.1	9.1	0.57	0.05	0.56	0.05
oxDNA2	G2-stiff	372	9.4	9.2	0.5	0.04	0.4	0.04
oxDNA2	G2-flex	366	9.1	8.8	0.4	0.07	0.4	0.07

Supporting Note 1

Oligonuclotides. DNA sequences for synthesizing the all-DNA G2-stiff and G2-flex dendrimers used in this study are listed in the following table. The bold letters correspond to sticky-end sequence, p indicates the position of the phosphate modification, T_4 and T_5 stands for a poly(thymine) sequence (in red) with length of four (4) and five (5) bases respectively and azide, dbco are the click-reactive groups (in blue) for performing the azide–cyclooctyne cycloaddition chemistry. The g_1 (core) and g_2 (shell) symbols in the name of the sequences indicate to which generation they belong.

Name	Sequences
Y_a-g_1-stiff	5'-p-**TGAC** TGGATCCGCATGACATTCGCCGTAAG-3'
Y_b-g_1-stiff	5'-p-**TGAC** CTTACGGCGAATGACCGAATCAGCCT-3'
Y_c-g_1-stiff	5'-p-**TGAC** AGGCTGATTCGGTTCATGCGGATCCA-3'
Y_a-g2-stiff	5'-p-**GTCA** TGGATCCGCATGACATTCGCCGTAAG-3'
Y_b-g2-stiff	5'-T5 CTTACGGCGAATGACCGAATCAGCCT-3'
Y_c-g2-stiff	5'-T5 AGGCTGATTCGGTTCATGCGGATCCA-3'
Y_a-g_1-flex	5'-azide-PEG4-TGGATCCGCATGACATTCGCCGTAAG-3'
Y_b-g_1-flex	5'-azide-PEG4-CTTACGGCGAATGACCGAATCAGCCT-3'
Y_c-g_1-flex	5'-azide-PEG4-AGGCTGATTCGGTTCATGCGGATCCA-3'
Y_a-g2-flex	5'-dbco-PEG4-TGGATCCGCATGACATTCGCCGTAAG-3'
Y_b-g2-flex	5'- T5 CTTACGGCGAATGACCGAATCAGCCT-3'
Y_c-g2-flex	5'- T5 AGGCTGATTCGGTTCATGCGGATCCA-3'

G2-stiff construction scheme. The Y-DNAs belonging to the first generation g_1 (Y-g_1-stiff) and second generation g_2 (Y-g_2-stiff) are assembled from three-partially complementary single-stranded DNAs at equal molar ratio.

Y-g_1-stiff = Y_a-g_1-stiff + Y_b-g_1-stiff+ Y_c-g_1-stiff

Y-g_2-stiff = Y_a-g_2-stiff + Y_b-g_2-stiff+ Y_c-g_2-stiff

To build up the G2-stiff dendrimer, three Y-g_2-stiff molecules are ligated to the sticky-ends of one Y-g_1-stiff molecule (G2-stiff = Y-g_1-stiff + 3 x Y-g_2-stiff).

G2-flex construction scheme. In similar fashion, the Y-DNAs belonging to the first generation g_1 (Y-g_1-flex) and second generation g_2 (Y-g_2-flex) are:

Y-g_1-flex = Y_a-g_1-flex + Y_b-g_1-flex + Y_c-g_1-flex

Y-g_2-flex = Y_a-g_2-flex + Y_b-g_2-flex+ Y_c-g_2-flex

To build up the G2-sflex dendrimer, three Y-g_2-flex molecules are reacted with one Y-g_1-flex molecule (G2-flex = Y-g_1-flex + 3 x Y-g_2-flex).

Supporting Note 2

Bead-spring (BS) model simulations. Both G2-stiff and G2-flex are modelled representing each branch of Y-unit as a string of charged monomers that interact trough potentials with parameters chosen to mimic equilibrium properties of dsDNA chain, as it was introduced in [1,2] and applied for the first 7 generations of all-DNA dendrimers in [3]. Steric interaction is modelled with truncated and shifted Lennard-Jones potential that reproduces the size of DNA helix diameter. Harmonic bonding potential is applied to preserve the equilibrium bond length between DNA base-pairs, while the stiffness of DNA is modelled with harmonic bending angle potential using 4 values for the constant of bending energy k_{ϕ}: $750kJmol^{-1}$ that recovers the persistence lengths of dsDNA chain, $150kJmol^{-1}$ is assigned to single stranded non-sticky ends, while $75kJmol^{-1}$ is applied to 8-bases long single-stranded subgeneration bridges in G2-flex and $0kJmol^{-1}$ stands for no energy penalty for rotations at the junction points. Electrostatic interaction is treated with Yukawa screening potential, since both molecules are studied in the solution of high salt concentrations. All BS model simulations are performed using ESPResSo package [4,5]. Simulations were run in canonical *NVT* ensembles with periodic boundary conditions at temperature T = 298 K using a Langevin thermostat. Approximately the first 10% of the samples were discarded in equilibration, depending on the convergence of quantities of interest.

Effective interactions: A single G2 all-DNA is simulated for 500 ns at a monomer density of $5.0 * 10^{-8}\text{Å}^{-3}$ to get the uncorrelated configurations. Then $5 * 10^{5}$ Widom insertions are performed for the calculation of the effective potential.

All monomer resolved simulations: In simulation box of size $9.05 * 10^{2}\text{Å}$, the simulation parameters (the number of simulation steps N_{steps} and total simulation time t_{tot}) at the packing fractions $\eta = 0.5$, $\eta = 1$, $\eta = 2$ are $N_{steps} = 9 * 10^{8}, 4 * 10^{8} and 1.5 * 10^{7}$ and $t_{tot} = 4.5 * 10^{-6}s, 2 * 10^{-6}s\ and\ 3.75 * 10^{-6}s$ respectively.

References

[1] A. Wynveen, C. N. Likos, *Phys. Rev. E* **2009**, 80, 1 010801.

[2] A. Wynveen, C. N. Likos, *Soft Matter* **2010**, 6, 1 163.

[3] C. Jochum, N. Adžić, E. Stiakakis, T. L. Derrien, D. Luo, G. Kahl, C. N. Likos, *Nanoscale* **2019**, 11 1604.

[4] H.-J. Limbach, A. Arnold, B. A. Mann, C. Holm, *Comp. Phys. Commun.* **2006**, 174, 9 704.

[5] A. Arnold, O. Lenz, S. Kesselheim, R. Weeber, F. Fahrenberger, D. Roehm, P. Košovan, C. Holm, Meshfree methods for partial differential equations VI. Springer, **2013** 1-23.
